# Œsophageal Pouches

**Published:** 1903-07-25

**Authors:** 


### ŒSOPHAGEAL POUCHES.

MR. BUTLIN[1] again directs attention to the uncommon but very interesting abnormality known as pressure-pouch of the œsophagus and shows that the diagnosis is comparatively easy, and operative treatment, on the whole, satisfactory. His conclusions are based upon eight cases on which he has operated and eight others where there was no reasonable doubt about the diagnosis. The symptoms are :—(1) Regurgitation of undigested food many hours or even a day or two after it has been taken. (2) Gurgling up of gas from the throat, particularly when pressure is made low down upon the left side of the neck, with, in some cases, a distinct bulging in this region. (3) Arrest of a bougie about nine inches from the teeth. Other occasional symptoms are wasting, pressure symptoms and the possibility of introducing a full-sized bougie into the stomach. With regard to the operation Mr. Butlin's experience is that it leads to a speedy, complete, and permanent cure of the disease, and is not fraught with more danger to life than the condition justifies. In order to remove the pouch an incision is made along the anterior border of the sterno-mastoid, the larynx is rotated to the right, and the pouch exposed. The pouch is then removed, and the wound in the pharynx sutured. A soft rubber tube is inserted down to the wound in the pharynx and the skin incision is not completely closed, the reason for this being that the wound in the pharynx always gives way so that some of the food taken passes out through the wound in the neck. This, however, does not continue for long, as the sinus in the neck heals up in a few weeks.

[1] British Medical Journal, July 11th.

